# USP15 Drives NSCLC Progression and Chemoresistance, Potentially via Regulation of the U2‐Type Spliceosomal Complex

**DOI:** 10.1002/cam4.71055

**Published:** 2025-08-05

**Authors:** Chien‐Chih Chiu, Sheng‐Kai Hsu, Wangta Liu, I‐Ling Lin, Wenhua Qiu, Ching‐Hung Hsieh, Chon‐Kit Chou

**Affiliations:** ^1^ Department of Biotechnology Kaohsiung Medical University Kaohsiung Taiwan; ^2^ Department of Biological Sciences National Sun Yat‐Sen University Kaohsiung Taiwan; ^3^ Department of Medical Research Kaohsiung Medical University Hospital Kaohsiung Taiwan; ^4^ Department of Medical Laboratory Science and Biotechnology Kaohsiung Medical University Kaohsiung Taiwan; ^5^ Center for Cancer Research, Kaohsiung Medical University Kaohsiung Taiwan; ^6^ Department of Laboratory Medicine Kaohsiung Medical University Hospital Kaohsiung Taiwan; ^7^ Institute of Chinese Medical Sciences, State Key Laboratory of Mechanism and Quality of Chinese Medicine University of Macau, Avenida da Universidade Taipa Macau P. R. China

**Keywords:** cancer progression, non‐small‐cell lung cancer, spliceosome, U2‐type spliceosomal complex, ubiquitin‐specific processing protease 15, USP15

## Abstract

**Objective:**

Non‐small cell lung cancer (NSCLC) is an aggressive and lethal malignancy with the highest cancer‐related mortality rate. More than 50% of patients are diagnosed at advanced stages, often accompanied by chemoresistance and poor prognosis. Deubiquitinases (DUBs), which regulate various signaling pathways by removing ubiquitin moieties, are frequently dysregulated in tumors, including the ubiquitin‐specific processing protease 15 (USP15). However, the biological role of USP15 in NSCLC progression remains poorly defined. This study aimed to investigate the biological function and mechanistic relevance of USP15 in NSCLC.

**Method:**

USP15 expression in human NSCLC tumor tissues and matched adjacent normal tissues was assessed by immunohistochemical staining. Western blotting was performed to evaluate USP15 protein levels in various NSCLC cell lines. Functional assays were conducted to examine the effects of USP15 knockdown on NSCLC cell growth, invasion, and epithelial–mesenchymal transition (EMT). Chemosensitivity assays were carried out using topotecan and irinotecan. Additionally, proteomic analysis was performed through immunoprecipitation of tGFP‐tagged USP15 followed by LC–MS/MS to identify USP15‐interacting proteins.

**Results:**

USP15 was significantly overexpressed in NSCLC tumor tissues compared to adjacent normal tissues, as confirmed by immunohistochemistry. This finding was further supported by western blot analysis showing elevated USP15 levels across multiple NSCLC cell lines. Knockdown of USP15 impaired NSCLC cell growth and invasion, reduced EMT marker expression, and re‐sensitized cells to topotecan and irinotecan. Proteomic profiling identified USP15‐interacting proteins enriched in the U2‐type spliceosomal complex and RNA helicase activity, suggesting a role for USP15 in regulating pre‐mRNA splicing.

**Discussion:**

This study demonstrates the oncogenic role of USP15 in NSCLC, highlighting its contribution to tumor progression, chemoresistance, and RNA processing. The findings provide mechanistic insights into how USP15 may drive NSCLC pathogenesis through modulation of spliceosome‐associated proteins. These results support the potential of USP15 as both a diagnostic biomarker and a therapeutic target in NSCLC.

## 1 Introduction

Lung cancer represents the most lethal cancer type, with non‐small‐cell lung cancer (NSCLC) accounting for approximately 85% of cases and showing a high tendency to metastasize to the brain, bone, and liver [1, 2, 3]. Over half of NSCLC patients are diagnosed at advanced stages, for which chemotherapy remains the standard treatment [4], including the use of topoisomerase I inhibitors such as topotecan and irinotecan, which exert their antitumor effects by inducing DNA damage [4]; however, their long‐term efficacy is often limited by the development of resistance through mechanisms such as impaired apoptotic signaling, enhanced drug efflux, and increased DNA repair activity [5, 6]. Even after curative resection, approximately 30%–50% of patients relapse or die due to disease recurrence [7]. The poor prognosis of NSCLC patients is attributed primarily to the lack of early diagnostic tools and effective therapeutic strategies for advanced disease. Hence, a better understanding of the molecular changes involved in NSCLC tumorigenesis is urgently needed.

Ubiquitination is a highly orchestrated posttranslational modification in which ubiquitin is covalently attached to substrate proteins, playing a crucial role in regulating protein turnover, signaling, and cellular homeostasis. This process is counteracted by deubiquitination, a reversible mechanism that removes ubiquitin moieties from protein substrates and is conducted by a group of deubiquitinating enzymes (DUBs) [8]. Human DUBs are broadly classified into two major classes: cysteine proteases and metalloproteases. The cysteine protease class includes six families—ubiquitin‐specific proteases (USPs), motif interacting with Ub‐containing novel DUBs (MINDYs), Machado–Joseph disease protein domain proteases (MJDs), ovarian‐tumor proteases (OTUs), ubiquitin carboxy‐terminal hydrolases (UCHs), Zn‐finger and UFSP domain proteins (ZUFSP), and monocyte chemotactic protein‐induced proteins (MCPIPs)—while the JAMM/MPN domain‐associated metallopeptidases (JAMMs) constitute the only family of metalloproteases [9]. Among them, USPs constitute the largest subfamily and are frequently dysregulated in various pathological conditions, particularly cancer [10, 11]. For example, USP35 is overexpressed in gastric cancer and facilitates metastasis by stabilizing Snail1 [12]. Notably, ubiquitin‐specific protease 15 (USP15) is of great interest because of its reported role in promoting NSCLC tumorigenesis [13].

In this study, we investigated the role and underlying mechanisms of USP15 in NSCLC tumorigenesis. Analysis of the TCGA database revealed that elevated USP15 expression is correlated with poor overall survival and is preferentially upregulated in several NSCLC cell lines. Genetic knockdown of USP15 suppressed NSCLC cell proliferation and invasion while enhancing chemosensitivity. To elucidate its molecular functions, we performed proteomic analysis to identify USP15‐associated binding partners, revealing enrichment in pathways related to the U2‐type spliceosomal complex and RNA helicase activity. Our findings suggest that USP15 promotes oncogenic processes in NSCLC, potentially by increasing the efficiency of pre‐mRNA splicing.

## 2 Materials and Methods

### 2.1 Cell Cultures

Five human non‐small‐cell lung cancer cell lines, namely, three adenocarcinoma cell lines (A549, CL1‐0, and H1437) and two large cell carcinoma cell lines (H1299 and H460), were used. Two nontumorigenic lung cell lines, the bronchial epithelial cell line BEAS‐2B and the fetal lung fibroblast line MRC‐5, were acquired from the American Type Culture Collection (ATCC; Manassas, VA, USA). The cell lines were cultured in DMEM/F12 (Gibco, Grand Island, NY, USA) supplemented with 2 mM glutamine, 8% fetal bovine serum (FBS), and 0.5% penicillin/streptomycin solution (Mediatech Inc., Herndon, VA, USA) at 37°C in a humidified atmosphere of 5% CO_2_. The cell lines used in the present study were confirmed to be free of Mycoplasma contamination via a PCR‐based assay.

### 2.2 Establishment of NSCLC Cell Lines With USP15 Knockdown

The oncogenic role of USP15 in vitro was evaluated under gene‐silencing conditions. USP15‐shRNA vectors were acquired from the National RNAi Core Facility (Academia Sinica, Taipei, Taiwan) and transduced into two lung adenocarcinoma cell lines, A549 and CL1‐0, to induce stable shRNA expression. The details are as follows: The shRNA sequence targeted the mRNA of human USP15 (sequence ID: NM_006313.1). The target sequence of the USP15 shRNA was 5′‐GATACAGAGCACGTGATTATT‐3′. The shRNA lentiviral expression construct pLKO.1‐puro was cotransfected into 293 T cells with pCMVdeltaR8.91 and pMD. G plasmids using TurboFect Transfection Reagent (Thermo Fisher Scientific). Viral supernatants were collected at 48, 72, and 96 h after transfection in DMEM/F12 supplemented with 8% FBS and filtered through Acrodisc syringe filters with a 0.45 μm Supor membrane to remove cell debris. The cells were transduced with the USP15‐shRNA vector and selected with puromycin for three weeks. The stably transfected cells were tested after transfection for 48 h.

### 2.3 Cell Growth Assays

Transfectants expressing low levels of USP15 were subjected to cell proliferation and colony formation assays. For the cell proliferation assay, the cells were plated in a 12‐well plate at 1 × 10^5^ cells per well and incubated at 37°C in a humidified atmosphere containing 5% CO_2_ for 48 h. After incubation, the cells were exposed to 0.2% trypan blue and counted by a Countess automated cell counter (Invitrogen, Carlsbad, CA, USA). Alternatively, to evaluate the ability of single cells to form colonies in vitro, a colony formation assay was performed. Briefly, the cells were placed onto six‐well plates at a concentration of 500 cells per well. The growth of the colonies was examined two weeks later via Giemsa staining.

### 2.4 Cell Migration and Invasion Assays

Cell migration and invasion are prerequisites for cancer metastasis, reflecting a greater degree of malignancy. Moreover, the impact of USP15 knockdown on the acceleration of cell motility deserves investigation. This finding was revealed by a wound healing assay. Briefly, 2 × 10^5^ cells were seeded and grown in a six‐well plate until a full monolayer formed. The confluent layer of cells was further scratched with a P‐200 pipette tip to create a straight wound. After incubation at 37°C for 14 h, the wound closure areas were photographed and analyzed with the freeware “TScratch.” To quantitatively evaluate the impact on cell invasiveness, a Matrigel invasion assay was conducted using a 24‐well BioCoat Matrigel invasion chamber (BD Biosciences, San Diego, CA). In the experiment, a total of 5 × 10^4^ cells were seeded into the upper chamber of the culture media, which was serum‐free. The lower chamber was filled with medium containing 10% FBS, which served as a chemoattractant. The cells were permitted to migrate for 14–20 h, and the invading cells (those that traversed the chamber) were immobilized with 0.5% formaldehyde and subjected to staining with Giemsa stain. The quantification of invaded cells was carried out via bright‐field microscopy at a magnification of 100×.

### 2.5 Western Blotting Assay

Whole‐cell lysates were prepared on ice with RIPA buffer containing 50 mM Tris–HCl (pH 7.5), 1% NP‐40, 0.5% sodium deoxycholate, 0.1% SDS, and 0.15 M NaCl. Equal amounts of total protein (30 μg of protein per lane) were separated via SDS–polyacrylamide gel electrophoresis (SDS–PAGE) on 8%–12% gels and subsequently transferred to polyvinylidene fluoride (PVDF) membranes.

The PVDF membrane was initially blocked with Tris‐based saline‐0.5% Tween 20 (TBS‐T) buffer containing 5% skim milk. The membrane was subsequently incubated with primary antibodies specific to the target proteins, followed by the corresponding secondary antibodies. Following thorough washing with TBS‐T, the protein bands were visualized via a chemiluminescence kit (Advansta Corp., Menlo Park, CA, USA) in accordance with the manufacturer's instructions.

### 2.6 Identification of USP15‐Binding Proteins via IP–MS/MS

Since an expression vector that expresses the USP15‐tGFP fusion protein is commercially available (origene, Cat. No. RG222642), we used an anti‐tGFP antibody to immunoprecipitate the tGFP‐tagged USP15 binding complex from H1299 and H460 cells. The cells were harvested on ice by using Pierce IP lysis buffer (Thermo Scientific, Cat. No. 87788), and protein lysates (1 mg) from stable tGFP‐tagged USP15 lines were incubated overnight with Protein A/G beaded agarose (50 μL), which had been previously incubated with an anti‐tGFP antibody (10 μg) at 4°C for 3 h. The beads were subsequently washed multiple times with Pierce IP lysis buffer. The immunoprecipitates were subsequently eluted from the beads through boiling in sample buffer for 10 min and subjected to SDS–PAGE. Next, the proteins were further stained with Coomassie blue. The regions of the SDS–PAGE gel corresponding to molecular weights of 180–130 kDa, 130–100 kDa, 100–75 kDa, and 75–63 kDa were excised from each sample lane, individually digested, and analyzed by LC–MS/MS. The raw MS/MS data were searched via MASCOT and matched to protein sequences in the SwissProt database (UniProtKB/Swiss‐Prot, which was released in 2017_06). After the binding partners of USP15 were identified, the immunoprecipitates were analyzed via western blotting with anti‐USP15 and antibodies against candidate binding partners. tGFP‐expressing stable lines were used as the negative control. In addition, the impacted genes were subjected to analysis via Ingenuity Pathway Analysis (IPA, Ingenuity Systems, Redwood City, CA, USA) with the exported content version 70,750,971 (Release Date: 2021‐10‐22).

### 2.7 Immunofluorescence Staining

The experiment involved seeding a total of 5 × 10^4^ cells on a glass slide, which was then subjected to immersion in 67% nitric acid and treatment with either 2 μM or 5 μM topotecan for 24 h. The cells were subsequently secured with 4% paraformaldehyde diluted in PBS for 5 min, which was ensured by permeabilization with 0.5% Triton X‐100 in PBS for 10 min. The fixed cells were then blocked with 1% bovine serum albumin (BSA) for 1 h and incubated with a primary antibody against phospho‐histone H2A. X (γH2AX) (sc‐101,696) at 4°C overnight. Following three washes with 1% BSA, the cells were labeled with FITC‐conjugated secondary antibodies (GeneTex, GTX26816) at 4°C for 1 h. Finally, the nuclei were dyed with 4′,6‐diamidino‐2‐phenylindole (DAPI) for 5 min. Images were taken via fluorescence microscopy (model TE2000‐U; Nikon, Tokyo, Japan).

### 2.8 Tissue Microarray and Immunohistochemistry (IHC) Analysis

The study utilized a human lung cancer tissue microarray (AF‐LucSur2201, AiFang Biological) containing 80 primary lung tumor tissues (including both adenocarcinomas and squamous cell carcinomas) with 80 matched adjacent normal tissues. All cases were pathologically confirmed with documented differentiation status (high, moderate, and low grade) and associated survival data. USP15 protein expression was evaluated by IHC using a monoclonal antibody (Proteintech, #67557‐1‐Ig, Clone 1C9G3). The digital quantification of the staining intensity was performed via the Visiopharm software via the standardized H‐score method, which was calculated as follows: H‐Score = ∑(pi × i) = (percentage of weak intensity cells × 1) + (percentage of moderate intensity cells × 2) + (percentage of strong intensity cells × 3), where pi represents the percentage of positive cells at each intensity level (0–100%), and the staining intensity was graded on a 0–3 scale (0 = negative/no staining, 1 = weak/faint yellow, 2 = moderate/brown–yellow, 3 = strong/brown). The H score ranged from 0 to 300, with higher values indicating stronger cumulative staining intensity. All slides were independently evaluated by two board‐certified pathologists who were blinded to the sample origins and clinical outcomes to ensure objective scoring.

### 2.9 Statistical Analysis

All the data are expressed as the mean ± standard deviations (SDs). Statistical analysis of the differences between the experimental and vehicle control groups was conducted via one‐way ANOVA using SigmaPlot v12 (Systat Software Inc., Point Richmond, California, USA). A *p* value of less than 0.05 was considered statistically significant.

## 3 Results

### 3.1 USP15 Is Overexpressed in NSCLC and Predicts Poor Survival

**FIGURE 1:**
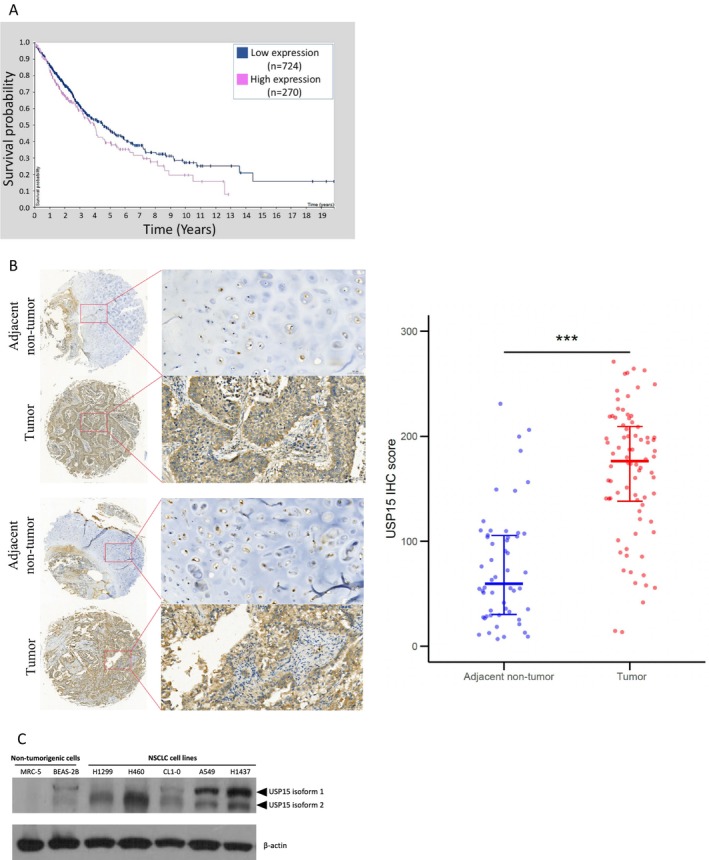
USP15 is highly expressed in NSCLC cells and is linked to NSCLC patient survival. (A) Kaplan–Meier survival analysis of lung cancer patients stratified by USP15 expression levels was performed via data from the Human Protein Atlas (HPA) database (https://www.proteinatlas.org). (B) Western blot analysis of the USP15 protein in a panel of human NSCLC and nontumorigenic cell lines. β‐Actin was used as an internal control. (C) IHC scores of USP15 expression in tumor tissues (*n* = 80) and matched adjacent nontumor tissues (*n* = 80) from a human lung cancer tissue microarray. Data are shown as medians with interquartile ranges. *p < 0.05, **p < 0.01, and ***p < 0.001 indicate statistically significant differences, as determined by the Wilcoxon rank‐sum test.

Numerous lines of evidence implicate USP15 as a key driver of tumor progression across multiple cancers. In hepatocellular carcinoma (HCC), its overexpression predicts early recurrence [14]; in breast cancer, it stabilizes ERα to accelerate disease progression [15]; and in bladder cancer, it activates NF‐κB signaling to promote tumor progression [16]. Here, we extended these observations to NSCLC and analyzed whether USP15 expression is correlated with NSCLC patient survival via data from the Human Protein Atlas database (https://www.proteinatlas.org/ENSG00000135655‐USP15/pathology/lung+cancer#imid_20029446). The results revealed that high USP15 expression is significantly associated with poor patient survival (Figure 1A), which is consistent with prior research linking elevated USP15 levels to unfavorable prognosis in patients with NSCLC [13, 17]. To validate these findings in clinical samples, we performed immunohistochemical (IHC) analysis of a tissue microarray comprising 80 pairs of NSCLC tumors and adjacent nontumor tissues (Figure S1A). USP15 expression was significantly higher in tumor tissues than in matched normal tissues (Figure 1B), although no stage‐dependent trend emerged in this cohort (Figure S1B). Immunoblotting further confirmed that USP15 was upregulated in a panel of NSCLC cell lines, including BEAS‐2B bronchial epithelial cells and MRC‐5 fetal lung fibroblasts, compared with nontumorigenic controls (Figure 1C). Collectively, these data identify USP15 as a tumor‐enriched protein in NSCLC and support its potential as a prognostic marker.

### 3.2 Knockdown of USP15 Inhibits the Proliferation of NSCLC Cell Lines

**FIGURE 2:**
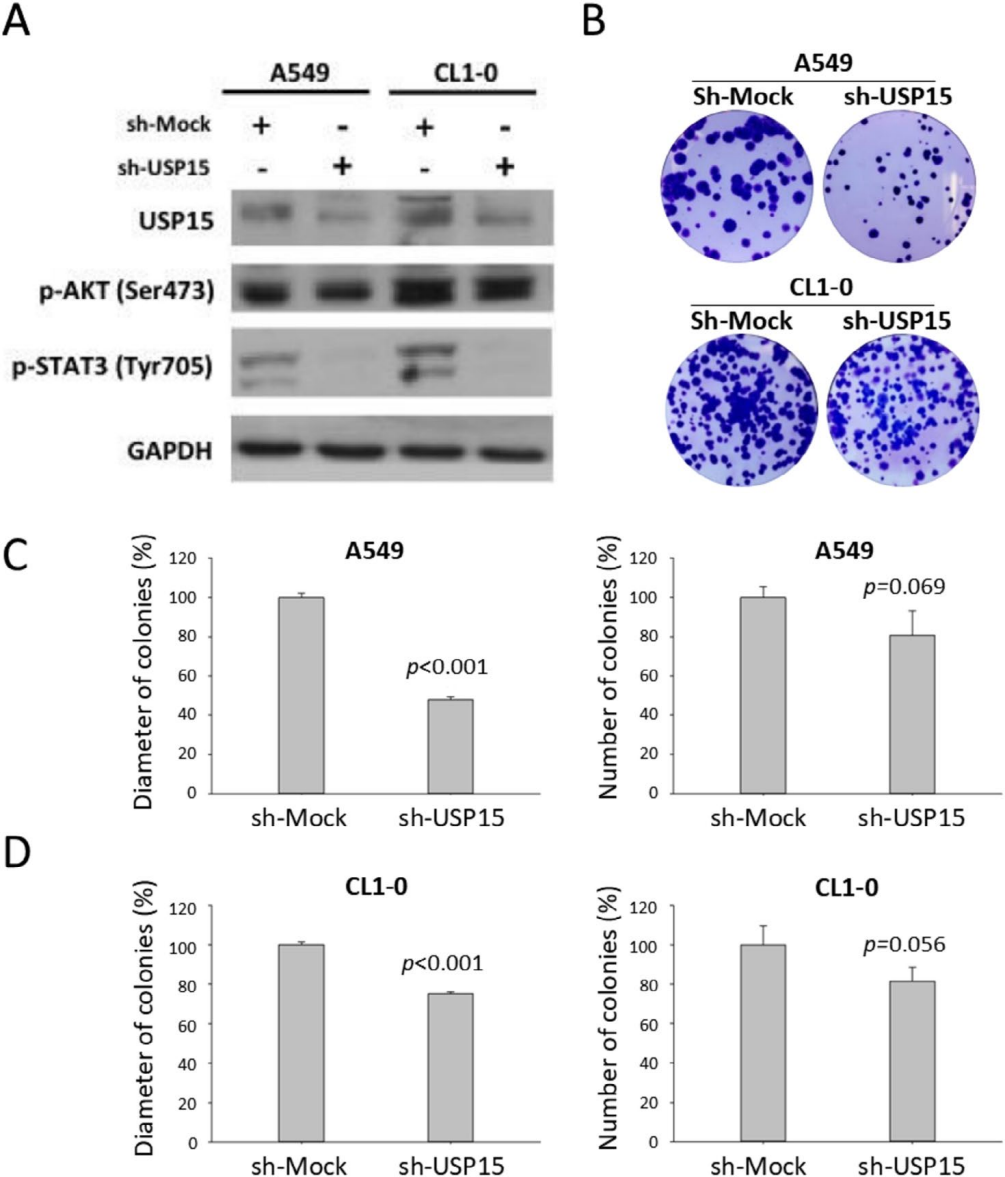
The effects of USP15 knockdown on the long‐term proliferation of NSCLC cells. A549 and CL1‐0 cells were stably transfected with a lentiviral vector expressing a shRNA targeting the human USP15 gene. (A) WB analysis verified the effectiveness of USP15 knockdown in the CL1‐0 and A549 cell lines. Furthermore, decreases in p‐AKT and p‐STAT3 were observed in USP15‐knockdown cells. GAPDH was used as an internal control. (B) The long‐term proliferation of A549 and CL1‐0 cells with or without USP15 knockdown was assessed via a colony formation assay. (C) and (D) Quantitative analysis of (B) the colony formation assay results. The data are presented as the means of three independent experiments. sh‐Mock: Mock control. sh‐USP15: USP15‐knockdown cells.

A large body of evidence has revealed the role of USP15 in tumorigenesis. However, reports regarding the role of USP15 in NSCLC are somewhat inconsistent. High expression of USP15, along with its close paralog USP4, has been reported to promote lung cancer cell proliferation [17]. In addition, the correlation between high USP15 expression and poor prognosis in NSCLC patients has been attributed to the upregulation of the matrix metalloproteinases MMP3 and MMP9 [13]. In contrast, another study reported that CRISPR/Cas9‐mediated knockout of USP15 in both the A549 and H1299 cell lines promoted cell migration and invasion [18]. Hence, the role of USP15 as either an oncogene or a tumor suppressor in NSCLC remains an intriguing area of study. We used a lentiviral RNAi approach to stably knock down USP15 in two NSCLC cell lines, A549 and CL1‐0, and confirmed the knockdown efficiency via western blotting (Figure 2A). Given that phosphorylated STAT3 (p‐STAT3) and AKT (p‐AKT) are frequently dysregulated in proliferating NSCLC cells [19, 20], we also examined their levels and found that USP15 knockdown significantly reduced both p‐STAT3 and p‐AKT expression (Figure 2A). Functionally, colony formation assays demonstrated that USP15 knockdown markedly impaired the clonogenic capacity of both cell lines (Figure 2B–D).

### 3.3 Knockdown of USP15 Suppresses Cell Invasion and Leads to Partial Loss of Mesenchymal Phenotypes

**FIGURE 3:**
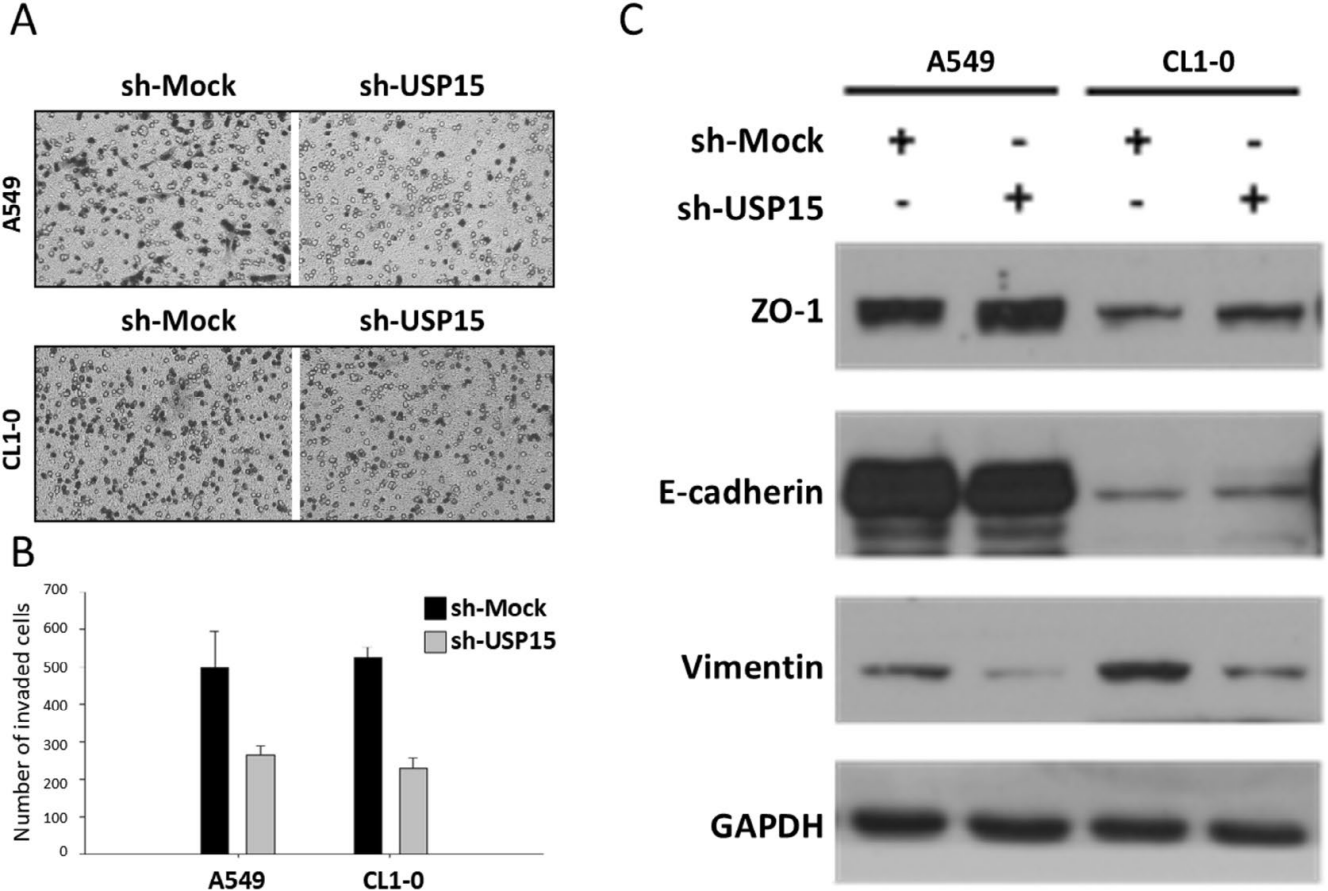
Knockdown of USP15 decreased invasion and partially reversed the EMT phenotype. (A) Effects of USP15 knockdown on both A549 and CL1‐0 cell invasion. A total of 1 × 10^5^ transfected cells were seeded on the upper layer of the Boyden chamber membrane, allowing the cells to penetrate the adjacent layer of the membrane. After 14 h, the invading cells were fixed with 4% paraformaldehyde and stained with Giemsa stain. (B) Quantitative analysis of (A) the invasion assay results. (C) Western blot analysis of A549 and CL1‐0 cells with USP15 knockdown revealed upregulation of the epithelial marker ZO‐1 but no changes in E‐cadherin expression. Moreover, there was a concomitant decrease in the expression of the mesenchymal marker vimentin.

Metastasis remains the leading cause of cancer‐related death [21]. A critical early step in this process is EMT, during which cancer cells lose their epithelial characteristics and acquire mesenchymal cell‐like properties, thereby gaining the ability to invade adjacent normal tissue [22]. Given that several members of the USP family have been shown to promote cancer metastasis when dysregulated [23, 24, 25], we investigated whether USP15 contributes to NSCLC cell invasiveness. Using a transwell invasion assay, we found that stable USP15 knockdown significantly impaired cell invasion compared with that of control cells (Figure 3A,B). We also speculated that USP15 knockdown may inhibit EMT. To test this hypothesis, we evaluated the protein levels of epithelial markers such as Zonula occludens‐1 (ZO‐1) and E‐cadherin, as well as the mesenchymal marker vimentin, via western blot analysis. Intriguingly, USP15 knockdown led to the upregulation of ZO‐1, a critical tight junction component, while E‐cadherin levels remained unchanged. Furthermore, the expression of the mesenchymal marker vimentin was notably reduced in USP15‐knockdown cells (Figure 3C), which is consistent with prior reports in gastric cancer and glioblastoma [26, 27]. Taken together, these findings suggest that downregulation of USP15 potentially reverses EMT and suppresses NSCLC cell invasion.

### 3.4 Knockdown of USP15 Sensitizes NSCLC Cells to Cytotoxic Agents by Reversing the Antiapoptotic Effect

**FIGURE 4:**
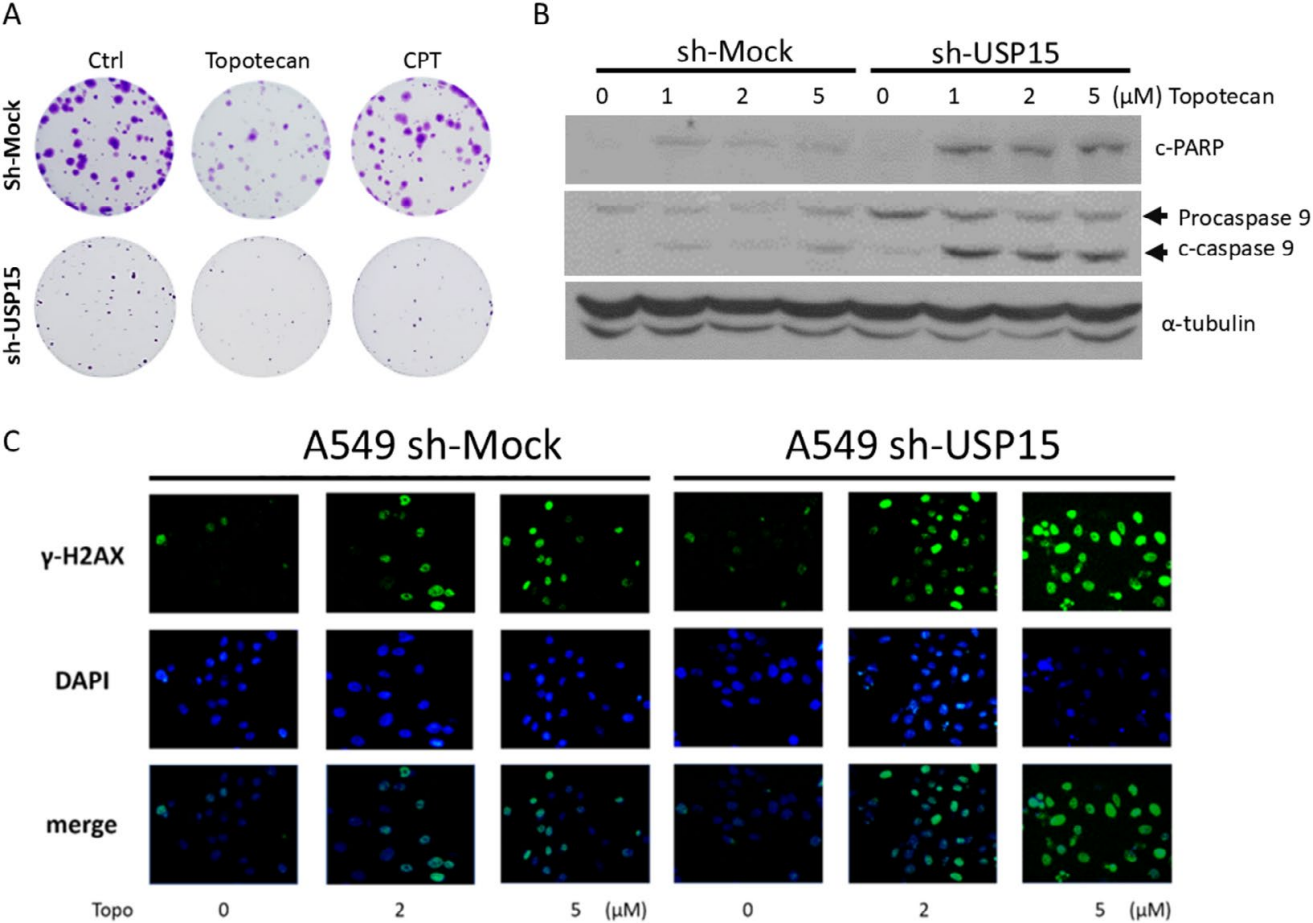
Knockdown of USP15 sensitizes NSCLC cells to DSB‐inducing agents. (A) NSCLC A549 cells were treated with the indicated doses of topotecan (Topo), a topoisomerase inhibitor, and long‐term proliferation and colony formation ability were determined via a colony formation assay. After 7 days, the cells were fixed with paraformaldehyde, followed by staining with Giemsa dye. Ctrl, vehicle control; Topo: Topotecan (0.025 μM) and CPT (0.01 μM). (B) Western blot results showing that topotecan, a topoisomerase inhibitor, may contribute to the chemoresistance of NSCLC cells, especially DNA damage inducers such as topotecan and irinotecan. DAPI was used to stain the nuclei. Drug treatment: 5 μM topotecan for 24 h. c‐PARP indicates the cleaved form of PARP, and c‐caspase 9 indicates cleaved caspase‐9 (active form). α‐Tubulin was used as an internal control. (C) Immunofluorescence assays revealed that USP15 knockdown increased the expression of γH2AX, a marker of DNA damage induced by topotecan, in NSCLC A549 cells, suggesting that USP15, especially DNA damage inducers, may contribute to the chemoresistance of NSCLC cells. DAPI is a nuclear marker. Drug treatment: 5 μM topotecan for 24 h. Magnification, 200 × .

As mentioned previously, drug resistance poses a significant challenge for patients with advanced NSCLC [6]. Recent studies have demonstrated that the aberrant expression of USPs enhances chemoresistance. These findings indicate that strategies to target USPs that limit apoptosis or promote tumor cell growth hold great promise in enhancing chemosensitivity [28, 29]. Hence, to determine whether USP15 contributes to chemoresistance, we used two chemotherapeutic agents—camptothecin (CPT) and its derivative topotecan—to evaluate their cytotoxicity in USP15‐knockdown A549 cells compared with mock‐transfected cells. Moreover, we examined whether USP15 inhibition exerts long‐term chemotherapy‐elicited cytotoxic effects at low concentrations. A549 cells were then treated with low concentrations of topotecan (0.025 μM) and CPT (0.001 μM) for 7 days, after which colony formation ability was assessed. The results revealed that the proliferative capacity of A549 cells in the USP15‐knockdown group was strongly suppressed compared with that in the sh‐mock group (Figure 4A). These findings suggest that the knockdown of USP15 potently augments the antineoplastic activity of chemotherapy against NSCLC. Given that the acquisition of chemoresistance in NSCLC is partly attributed to enhanced antiapoptotic activity [5, 30], we hypothesize that USP15‐induced chemoresistance may be driven by the inhibition of apoptosis. Compared with those in the mock‐transfected control group, the levels of proapoptotic markers, such as cleaved caspase‐9 and cleaved PARP‐1, in the USP15‐knockdown group increased in a dose‐dependent manner (Figure 4B). These findings revealed that USP15 downregulation sensitizes A549 cells to topotecan. In addition, given that topotecan is a topoisomerase I inhibitor that can induce DNA damage [31], we also investigated whether USP15 knockdown enhances topotecan‐induced DNA damage in NSCLC, as evaluated by immunofluorescence analysis of the damage marker γ‐H2AX. The results revealed that the γ‐H2AX signal was stronger in sh‐USP15 A549 cells after exposure to topotecan, suggesting that DNA damage was promoted by USP15 downregulation (Figure 4C).

### 3.5 Identification of USP15‐Interacting Proteins and Their Involvement in Spliceosomal Dynamics

**FIGURE 5:**
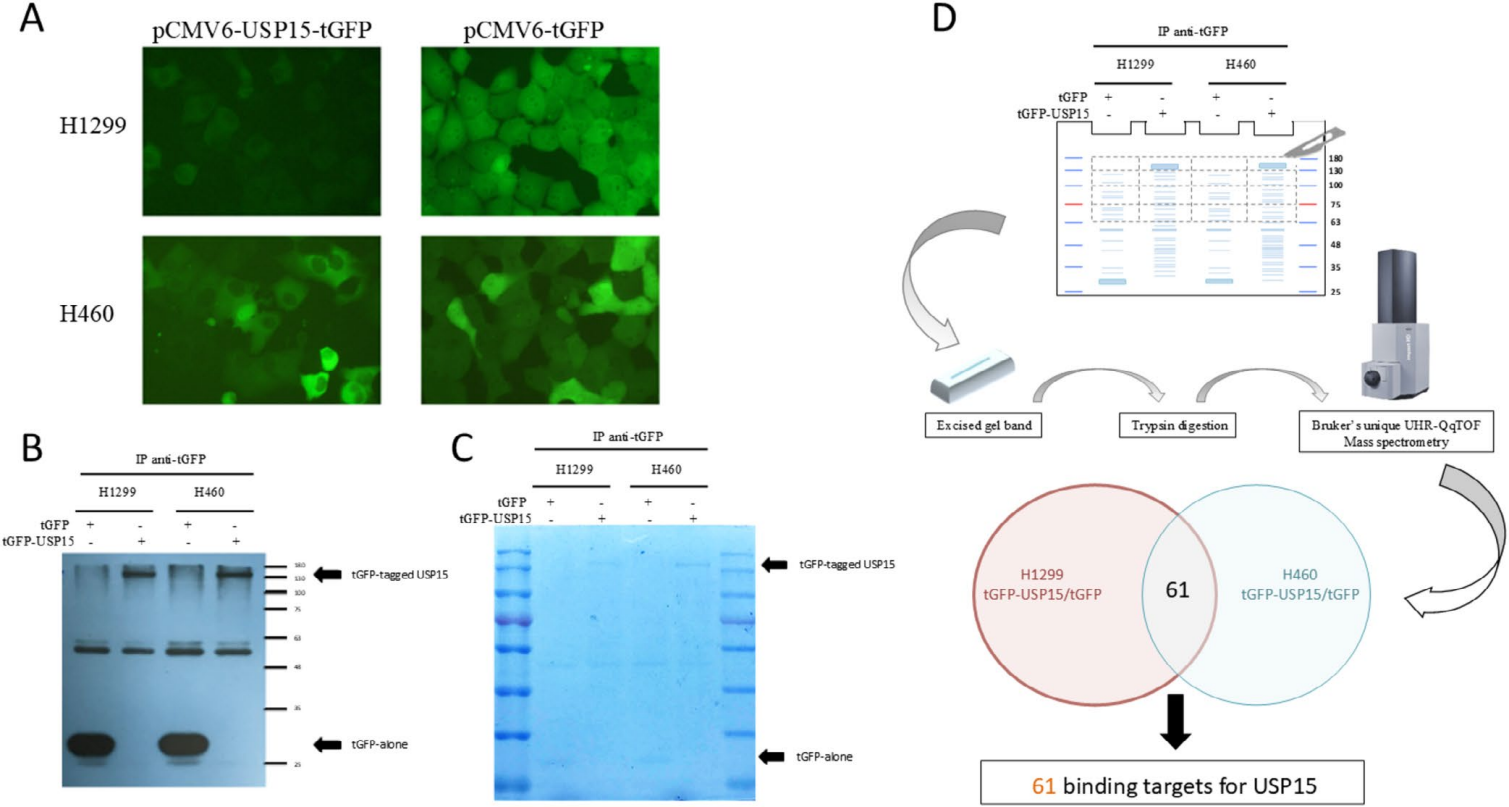
Flowchart of the identification of USP15‐associated proteins via IP–MS/MS. USP15‐associated proteins identified by IP–LC–MS/MS. (A) pCMV6‐USP15‐tGFP and pCMV6‐tGFP expression vectors were transfected into H199 and H460 cells, respectively, which express low levels of USP15. tGFP‐tagged USP15 is localized primarily in the cytosol. (B) Proteins were precipitated with an anti‐tGFP antibody. Bound proteins were detected with an anti‐tGFP antibody, confirming the precipitation of tGFP‐tagged USP15 in both H1299 and H460 cells (lanes 2 and 4). (C) Bound proteins were detected by Coomassie blue staining, which revealed that tGFP‐tagged USP15 was abundantly expressed in both H1299 and H460 cells (lanes 2 and 4). (D) Schematic diagram of the immunoprecipitation procedure and subsequent data analysis. Protein lysates were subjected to immunoprecipitation with monoclonal anti‐tGFP. The immunoprecipitates were separated via SDS–PAGE and detected via Coomassie blue staining. Excised gel pieces were digested and then analyzed via LC–MS/MS. We performed two proteomic studies examining the binding targets of USP15 after the immunoprecipitation of lysates from H1299 and H460 cells with monoclonal anti‐tGFP. We identified 61 proteins associated with USP15 in both NSCLC cell lines expressing tGFP‐tagged USP15 protein but not in the corresponding cells expressing tGFP alone.

Given that USP15 has deubiquitinating activity and performs its cellular functions through protein–protein interactions, we sought to identify its interacting partners. We established two NSCLC cell lines, H1299 and H460, expressing either TurboGFP (tGFP)‐tagged USP15 or tGFP alone as controls (Figure 5A). The cell lysates were subjected to immunoprecipitation with an anti‐tGFP antibody, and the pulled‐down complexes were separated via SDS–PAGE and further confirmed by western blotting with a monoclonal antibody against tGFP (Figure 5B) and Coomassie blue staining (Figure 5C). These findings confirmed the expression of the exogenous USP15‐tGFP fusion protein (with an estimated molecular weight between 180 and 130 kDa) in both H1299 and H460 cells. Four portions of the SDS–PAGE gel corresponding to molecular weights of 180–130 kDa, 130–100 kDa, 100–75 kDa, and 75–63 kDa were excised from each sample lane, digested individually, and analyzed by LC–MS/MS. The raw MS/MS data were searched via MASCOT and matched to protein sequences in the Swiss‐Prot 2017_06 database. To reduce the possibility of identifying nonspecific proteins, two filtering criteria were employed: (1) proteins detected in the cells expressing tGFP alone (negative control) were excluded; (2) the theoretical molecular weights of identified proteins did not differ from the molecular weights of the corresponding gel pieces.

**FIGURE 6:**
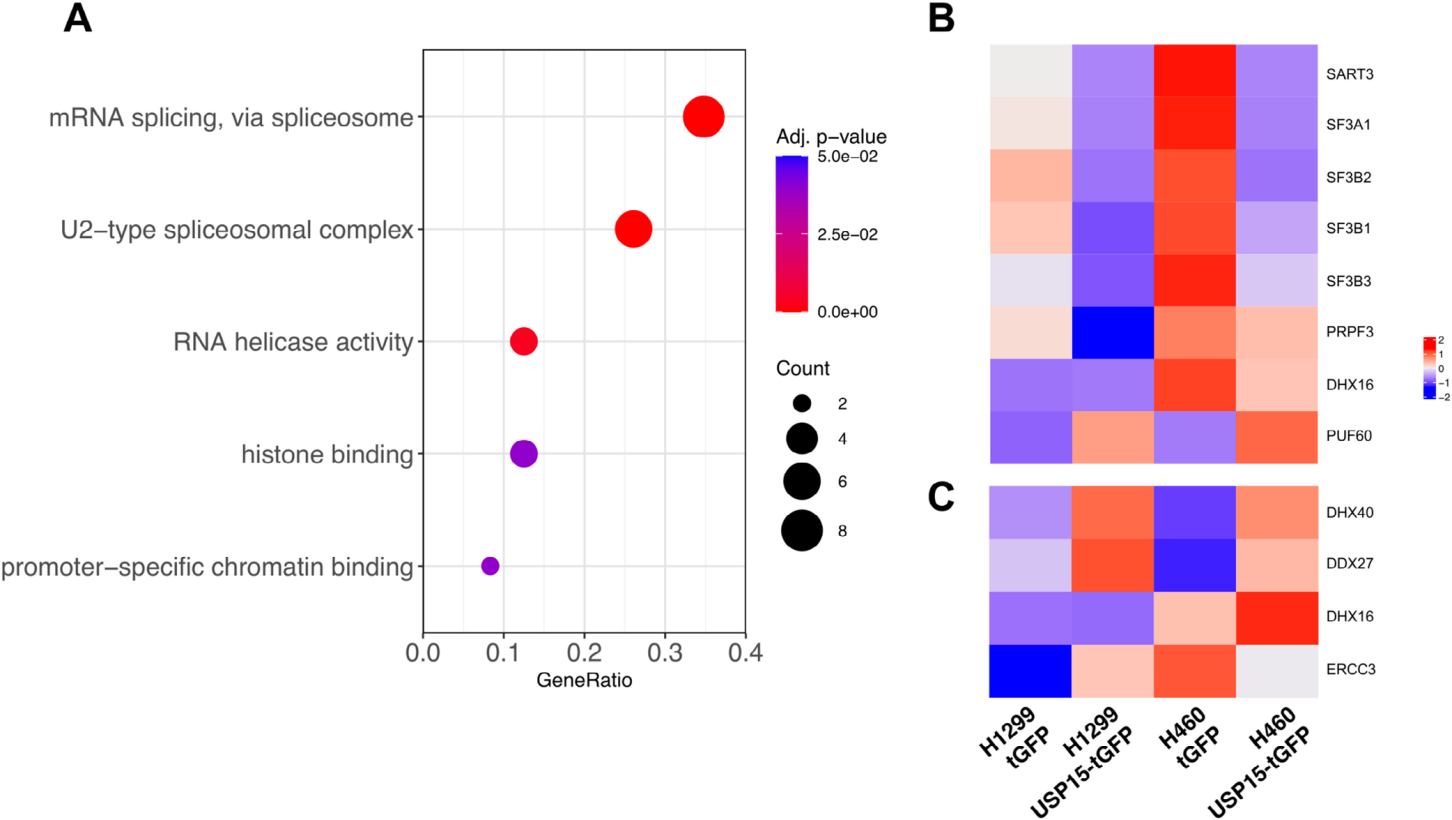
USP15‐associated proteins are enriched in U2‐type spliceosome components and RNA helicases. (A) Gene Ontology (GO) enrichment analysis of 61 high‐confidence USP15‐interacting proteins identified in two NSCLC cell lines stably expressing USP15‐tGFP compared with control lines expressing tGFP alone. The analysis revealed significant enrichment of proteins involved in the U2‐type spliceosomal complex and RNA helicase activity. (B) Heatmap showing the relative abundance of eight USP15‐associated proteins annotated as U2‐type spliceosome components: SART3, SF3A1, SF3B1, SF3B2, SF3B3, PRPF3, DHX16, and PUF60. (C) Heatmap displaying USP15‐associated proteins categorized as RNA helicases, including DHX40, DDX27, DHX16, and ERCC3.

Integration of the proteomic data from two NSCLC cell lines yielded 61 high‐confidence USP15‐associated proteins (Figure 5D), including known interactors such as squamous cell carcinoma antigen recognized by T cells 3 (SART3), a U4/U6 snRNP recycling factor [32], and novel candidates. Notably, approximately 16 of these proteins are involved in pre‐mRNA splicing (see Table S1; proteins involved in pre‐mRNA splicing are marked with an asterisk). Gene Ontology (GO) analysis revealed significant enrichment of proteins related to the U2‐type spliceosomal complex and RNA helicase activity (Figure 6A). A heatmap highlighted eight proteins—SART3, SF3A1, SF3B1, SF3B2, SF3B3, PRPF3, DHX16, and PUF60—annotated as U2‐type spliceosomal components (Figure 6B), whereas others, such as DHX40, DDX27, DHX16, and ERCC3, were categorized as RNA helicases (Figure 6C). Ingenuity pathway analysis (IPA) revealed that 16.3% of the USP15‐interacting proteins (8 out of 49) participate in the spliceosome cycle (Figure S2A,B). Of particular interest was splicing factor 3B subunit 1 (SF3B1), an essential component of the U2 small nuclear ribonucleoprotein (snRNP), which presented one of the highest Mascot scores. This finding aligns with the established role of USP15 in regulating snRNP assembly and spliceosome dynamics. As SF3B1 mutations have been linked to an impaired DNA damage response and chromosomal instability [33, 34], the USP15‐SF3B1 interaction may represent a functional axis influencing the response to DNA damage in NSCLC.

## 4 Discussion

The role of USP15 in cancer progression has been a subject of increasing interest in recent years. Our study provides insight into the function of USP15 in NSCLC, expanding upon previous research and revealing novel mechanisms of action. Our findings demonstrated that USP15 is significantly upregulated in NSCLC cell lines compared with normal lung cells. This upregulation is correlated with poor survival in NSCLC patients, which aligns with the conclusions drawn in prior research [13, 17]. These observations contrast with those of several earlier studies in other cancer types in which USP15 was reported to have tumor‐suppressive effects [18], highlighting the paradoxical role of USP15 in cancer progression.

Our knockdown experiments demonstrated that USP15 plays a crucial role in the proliferation and invasion of NSCLC cells. The observed decrease in colony formation ability and invasive potential upon USP15 knockdown suggested that USP15 functions as an oncogene rather than a tumor suppressor gene in NSCLC, promoting both tumor growth and metastatic potential. Additionally, our study provides novel insights into the role of USP15 in EMT, a critical process in cancer metastasis. USP15 knockdown resulted in the upregulation of the epithelial marker ZO‐1 and the downregulation of the mesenchymal marker vimentin. This finding is consistent with observations in GC and GBM [26, 27], suggesting that USP15 plays a conserved role in promoting EMT across different cancer types.

Moreover, the involvement of USP15 in the mechanisms of DNA repair has also been shown across various cancer types. For example, a study by Sun et al. reported that triple‐negative breast cancer cells rely on USP15‐dependent stabilization of PARP1, likely due to the absence of ER, PR, and HER2 receptors. This leads to elevated PARP1 levels and increased base excision repair, contributing to resistance to DNA damage‐inducing chemotherapy [35]. In addition, USP15 has been shown to play a critical role in homologous recombination (HR) repair by promoting the retention of BARD1/BRCA1 complexes at DNA double‐strand breaks (DSBs), a process essential for effective resolution of DNA damage [36]. Consistent with these observations, our study reveals a previously unrecognized role for USP15 in mediating chemoresistance to DNA damage‐inducing agents in NSCLC. Specifically, USP15 knockdown sensitized NSCLC cells to the clinical anticancer drugs CPT and topotecan, both of which induce DSBs. This increased sensitivity was accompanied by elevated DNA damage levels and the upregulation of proapoptotic markers. Moreover, we observed that USP15 knockdown resulted in the downregulation of p‐STAT3 and p‐AKT (Figure 2A), two signaling molecules known not only as oncogenic effectors but also as facilitators of DNA damage repair [37, 38]. Sustained activation of these two signaling molecules can confer resistance to genotoxic stress in NSCLC [39, 40]. However, whether USP15 knockdown‐induced chemosensitization is mediated through decreased p‐STAT3 and p‐AKT levels and whether these molecules act downstream or independent of USP15 remain to be elucidated. Overall, our findings align with emerging evidence linking USP15 overexpression to resistance to DNA damage‐inducing agents [41].

Through comprehensive proteomic analysis, we identified 61 candidate proteins that can interact with USP15 (Figure 5 and Table S1). Notably, several of these proteins, including SART3, a previously recognized USP15 binding partner, are involved in pre‐mRNA splicing [32]. GO and IPA revealed that the USP15‐associated proteins are predominantly enriched in the U2‐type spliceosomal complex and RNA helicase activity pathways (Figure 6). Among these, SART3 has been reported to facilitate lung tumorigenesis by modulating the mRNA splicing of CD44 [42]; SF3A1, a core spliceosomal component, has been implicated in the pathogenesis of HCC and colorectal cancer [43]; SF3B1, which is frequently mutated and overexpressed in NSCLC, can confer ferroptosis resistance via the upregulation of SLC7A11 [44]; and SF3B2 has been demonstrated to drive aggressive phenotypes of prostate cancer via the generation of the androgen receptor splice variant AR‐V7 [45]. Some studies further support the recognition of an interplay between the dysregulation of USPs, pre‐mRNA splicing, and the DNA damage response. For example, Ka et al. demonstrated that the spliceosomal component IK is required for ATM pre‐mRNA splicing and that its stabilization by USP47 is essential for efficient DNA repair [46]. Similarly, Tsao et al. reported that aberrant RNA methylation can trigger genotoxic stress through the recruitment of repair complexes via the ASCC‐ALKBH3 pathway [47]. Moreover, Kim et al. reported that USP39 participates in the DNA damage response through spliceosome‐dependent regulation of both nonhomologous end joining (NHEJ) and homologous recombination (HR) repair [48]. Together, these findings underscore the relevance of some USP family members in the regulation of pre‐mRNA splicing and DNA repair, potentially linking splicing dysregulation to cancer progression and treatment resistance.

Although our findings regarding USP15 upregulation in NSCLC and its association with poor prognosis are in agreement with those of previous studies, they stand in contrast to the findings of Kim et al. [18], who reported that USP15 downregulation suppresses NSCLC progression by modulating the TRAF6‐BECN1 signaling axis. Their work demonstrated that USP15 knockout enhanced cancer cell migration and invasion and that USP15 attenuated autophagy through BECN1 deubiquitination. However, our data indicate that USP15 is upregulated in NSCLC cell lines and is correlated with poor patient survival. Functionally, we found that USP15 promotes both invasion and chemoresistance in NSCLC. Furthermore, our proteomic analysis revealed novel USP15 binding partners involved in spliceosome regulation, providing deeper insight into the multifaceted roles of USP15 in tumor biology. This discrepancy may reflect the context‐dependent nature of USP15 function, which could vary depending on the tumor subtype, cellular environment, or interaction partner. As discussed above, USP15 interacts with SART3, a factor known to increase the deubiquitinating activity of USP15 [32]. This interaction may stabilize oncogenic proteins, further promoting tumor progression. Thus, the cellular outcome of USP15 activity likely depends on the specific protein complexes it forms, which may dictate whether USP15 acts in an oncogenic or tumor‐suppressive manner. While our study provides insight into the role of USP15 in NSCLC, there are several limitations to the current study that should be addressed in future research. First, it is imperative to conduct in vivo studies to validate the impact of USP15 on tumor growth, metastasis, and chemoresistance. Second, the mechanisms by which USP15 regulates its binding proteins and influences signaling pathways require further elucidation. Finally, the potential of USP15 as a therapeutic target needs to be explored through the development and testing of specific inhibitors.

## 5 Conclusion

The intricate balance between ubiquitination and deubiquitination is essential for cellular homeostasis, and its disruption is closely associated with cancer progression. Our study identified USP15 as a key contributor to NSCLC proliferation, invasion, and chemoresistance. High USP15 expression was correlated with poor patient prognosis in TCGA datasets and was significantly elevated in NSCLC tissues compared with adjacent nontumor tissues, supporting its oncogenic role. Proteomic analysis also revealed that USP15 is intimately linked to the U2‐type spliceosomal complex and RNA helicase activity, suggesting its specific role in pre‐mRNA splicing. These findings highlight that USP15 promotes NSCLC progression and could be a promising therapeutic target for future drug discovery.

## Author Contributions

**Chien‐Chih Chiu:** funding acquisition, writing – original draft. **Sheng‐Kai Hsu:** writing – original draft, investigation, formal analysis. **Wangta Liu:** software. **I‐Ling Lin:** validation. **Wenhua Qiu:** formal analysis. **Ching‐Hung Hsieh:** data curation, writing – review and editing. **Chon‐Kit Chou:** methodology, data curation, supervision, funding acquisition, project administration, writing – review and editing, writing – original draft.

## Ethics Statement

The authors have nothing to report.

## Consent

The authors have nothing to report.

## Conflicts of Interest

The authors declare no conflicts of interest.

## Use of Artificial Intelligence Tools

We prepared the manuscript with AI assistance to improve its readability. All the references have been checked and validated by the authors. Neither the figures nor the tables were adjusted or modified via AI assistance.

## Supporting information


Figure S1.
Figure S2.

Table S1.

## Data Availability

The data that support the findings of this study are available from the corresponding author upon reasonable request.
