# Supplementary figures and images for: USP15 Drives NSCLC Progression and Chemoresistance, Potentially via Regulation of the U2‐Type Spliceosomal Complex
Source: Cancer Med. 2025 Aug 5;14(15):e71055. doi: 10.1002/cam4.71055 (PMC12322926; doi:10.1002/cam4.71055)

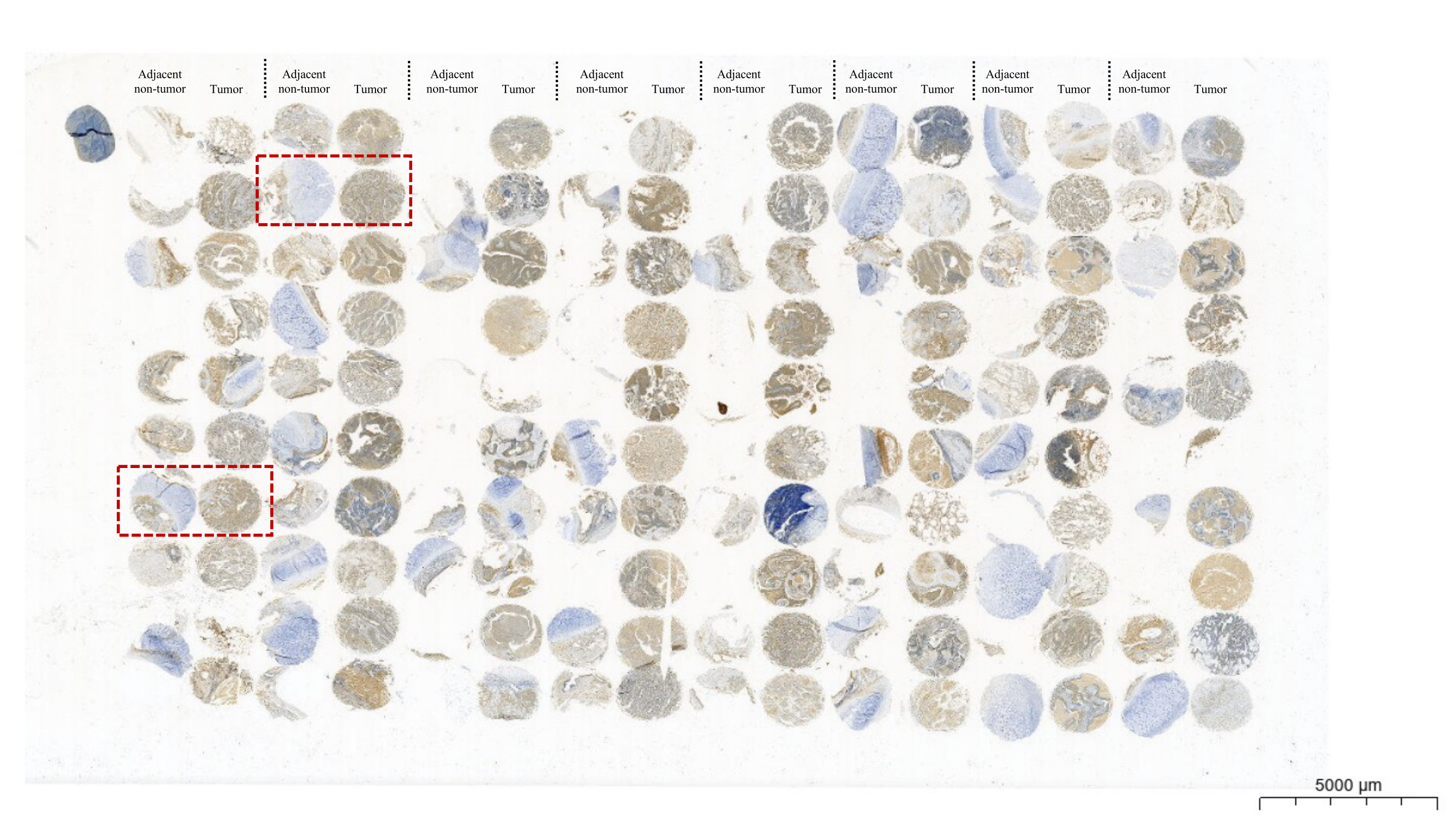

Supplement: Supplementary file 1 — Figure S1. Figure S2. [file CAM4-14-e71055-s001.zip › CAM4_71055_f1_Supplementary Fig-1A.tiff]

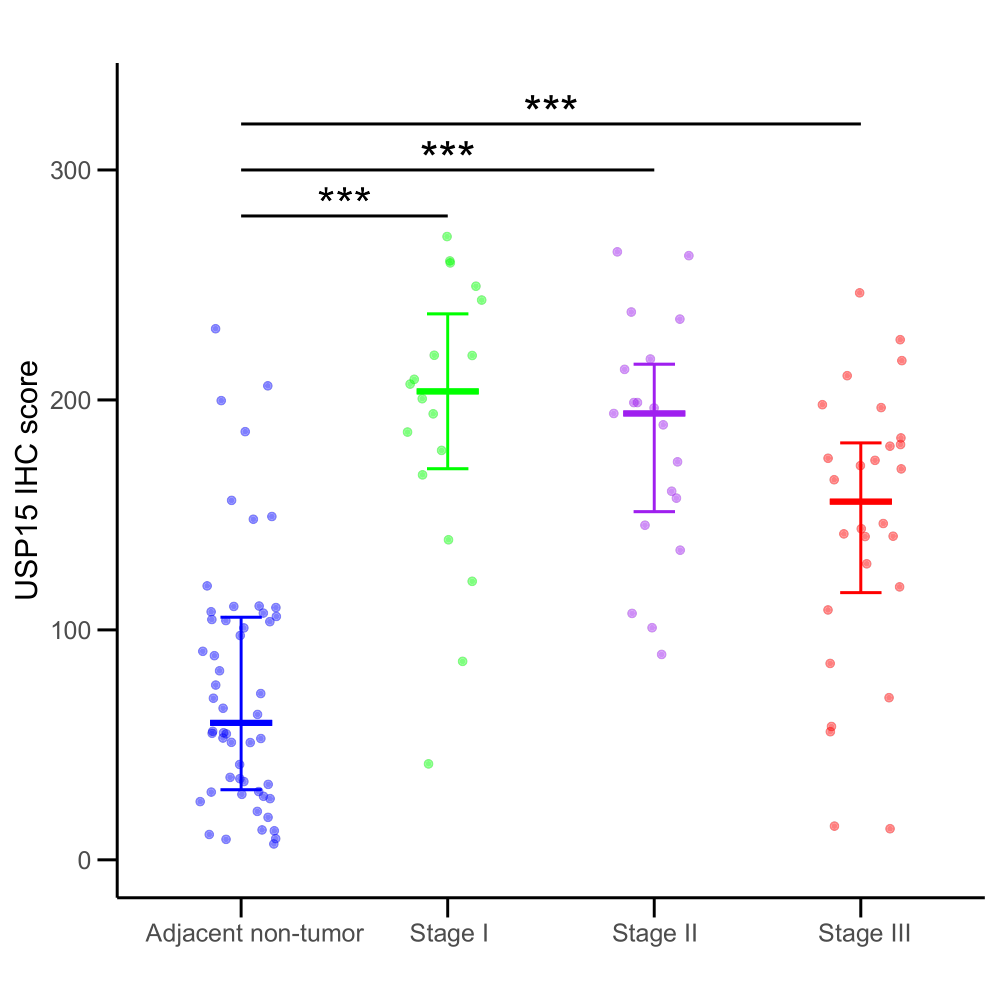

Supplement: Supplementary file 1 — Figure S1. Figure S2. [file CAM4-14-e71055-s001.zip › CAM4_71055_f1_Supplementary Fig-1B.tiff]

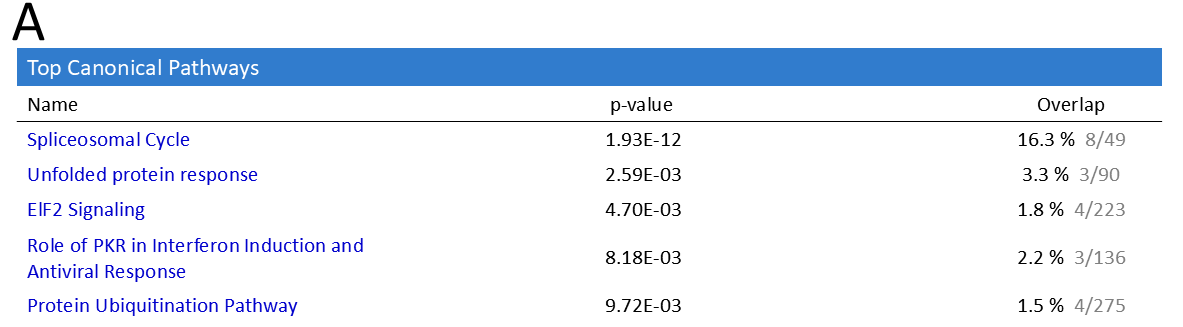

Supplement: Supplementary file 1 — Figure S1. Figure S2. [file CAM4-14-e71055-s001.zip › CAM4_71055_f2_Supplementary Fig-2A.tif]

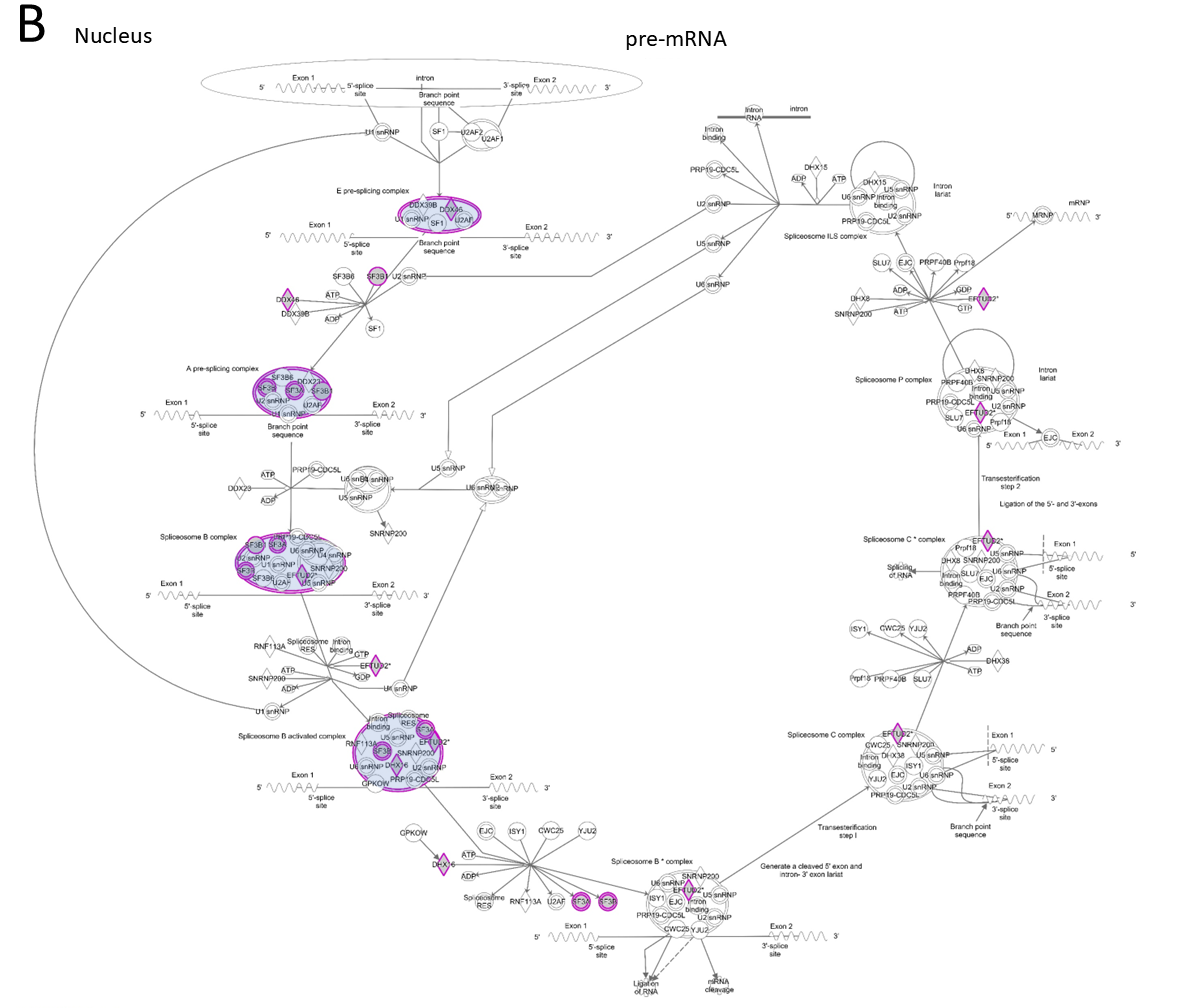

Supplement: Supplementary file 1 — Figure S1. Figure S2. [file CAM4-14-e71055-s001.zip › CAM4_71055_f2_Supplementary Fig-2B.tif]
